# The use of queueing theory for planning automated analytical systems

**DOI:** 10.1155/S1463924687000191

**Published:** 1987

**Authors:** T. L. Pap, L. Leisztner

**Affiliations:** 1 Institute of Analytical Chemistry University of Veszprém PO Box 158 Veszprém H-8201 Hungary; 2 Institute of Forensic Science PO Box 314/4 Budapest H-1903 Hungary


**Journal of Automatic Chemistry**/*Journal of Clinical Laboratory Automation*, **Vol. 9, No. 2 (April–June 1987), pp. 87–91**

### The use of queueing theory for planning automated analytical systems

**T. L. Pap**

*Institute of Analytical Chemistry, University of Veszprém, PO Box 158, H-8201 Veszprém, Hungary*

**and L. Leisztner**

*Institute of Forensic Science, PO Box 314/4, H-1903 Budapest, Hungary*

### Introduction

Queueing theory can be used in analytical chemistry if a large number of samples need to be analysed. It can be applied to optimizing the work organization of a real laboratory and for estimating performance and the utilization factor. However, most important application of this method is in the design of new laboratories.

For a laboratory design, the minimum number of analysis channels, the running time (the running time = waiting time + service time), the waiting time, the performance characteristics of a channel and so on, can be determined by the help of queueing theory if the distribution of arrival time and of service time and the priority of different samples are known.

This paper reports on the background to the development of an automated analytical system for headspace gas chromatographic analysis of some 50 000 bloodsamples/year.

All blood samples should be analysed by three indepoendent parallel measurements. This means that the samples are prepared by three different people and the chromatographic measurements are made on three different instruments. The laboratory was constructed on the basis of arrival information on blood samples over a five-year period. The running time of the samples, work-load and the fact that 10% of the measurements have to be repeated were taken into account in the design.

### Preliminaries

The first application of queueing theory was the design of telephone lines. One of the most popular monographs in this field was written by Kleinrock [1]. There is very little information in the literature concerning the use of queueing theory in analytical chemistry, although it is very useful in the design of an analytical system and in estimating its efficiency.

Adeberg and Doerffel [2] investigated average waiting time in terms of the number of service personnel and they established an optimum staffing level with queueing theory.

Massart *et al.* [3] investigated the M/M/l and M/M/n systems from queueing systems. They assumed that these systems are the most typical in analytical laboratories, where the distribution of both inter-arrival and service time are exponential. They discussed effects of the fluctuation of the analysis time, the number of channels, the number of service personnel, and the use of priorities on the waiting time.

Vollenbroek [4] also used the M/M/l model in the design of the working order of a laboratory for structural analysis (IR, MS, 1H-NMR, 13C-NMR) and produced a model simulating a real laboratory.

Vandeginste [5] has looked at the possibility of constructing a useful model to analyse the structure of a laboratory. The author dealt with data collection, data independence proof, construction of the model, the validity of the model, and the conclusions drawn from the queueing model.

### Design of the automated analytical system

The data shown in table 1 and the number of arrivals per working day with different priorities were used in the study. The distribution of the number of samples per working day are indicated in figure 1.

Distribution of inter-arrival and service time can be approximated with the help of a probability density function. The analytical solution may be used if the successive values of the probability variables (for instance the inter-arrival time intervals) are independent.

An independence test was performed on inter-arrival times with the mean square successive difference test [6]. The *D*-values of the tested years (table 1) were obtained from the following equation:

$$D = \frac{\dfrac{\sum_{i=1}^{v-1} (x_{i+1} - x_i)^2}{2(n-1)}}{\dfrac{\sum_{i=1}^{n} (x_i - \bar{x})^2}{n-1}} = \frac{\sum_{i=1}^{n-1} (x_{i+1} - x_i)^2}{2\sum_{i=1}^{n} (x_i - \bar{x})^2}$$

where $x_i$ = the number of samples arrived on the *i*th day
$\bar{x}$ = the mean value of samples in a working day
$n$ = the number of working days in a year.

The critical *D*-value is 0·99935 and this means that the sample arrival in the last five years was not independent (see table 1). So a numerical method based on the appropriate model of the laboratory was used instead.

*Table 1. Number of blood samples arriving for analysis and the D-values of the mean square successive difference tests in the years 1980–1984.*

| Year | Number of samples | D-value |
|---|---|---|
| 1980 | 39 500 | 0·82 |
| 1981 | 42 700 | 0·94 |
| 1982 | 43 300 | 0·82 |
| 1983 | 47 300 | 0·93 |
| 1984 | 48 100 | 0·88 |

The construction of a proper model of the laboratory is a very complicated task and it is difficult to control validity. The 'appropriate' model is simple enough for numerical calculation, but it adequately describes the laboratory and is capable of dealing with data which are not independent.

The work was divided into two steps. In the first stage, the laboratory as a whole was tested on the basis of the empirical sample arrival by the help of a black-box model

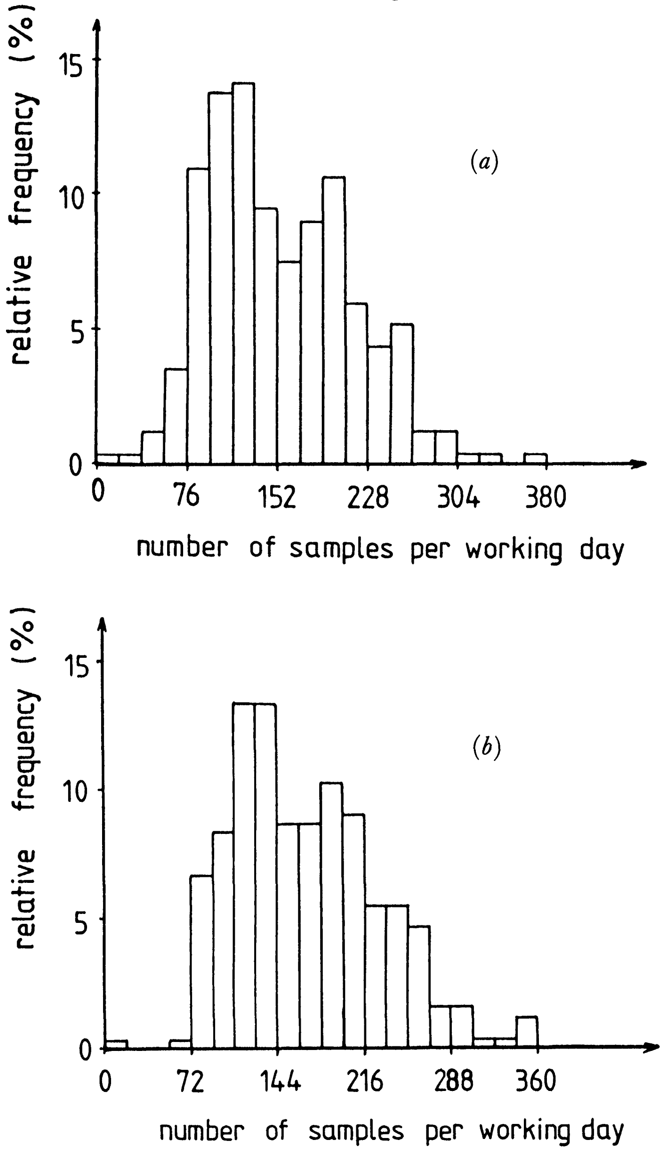

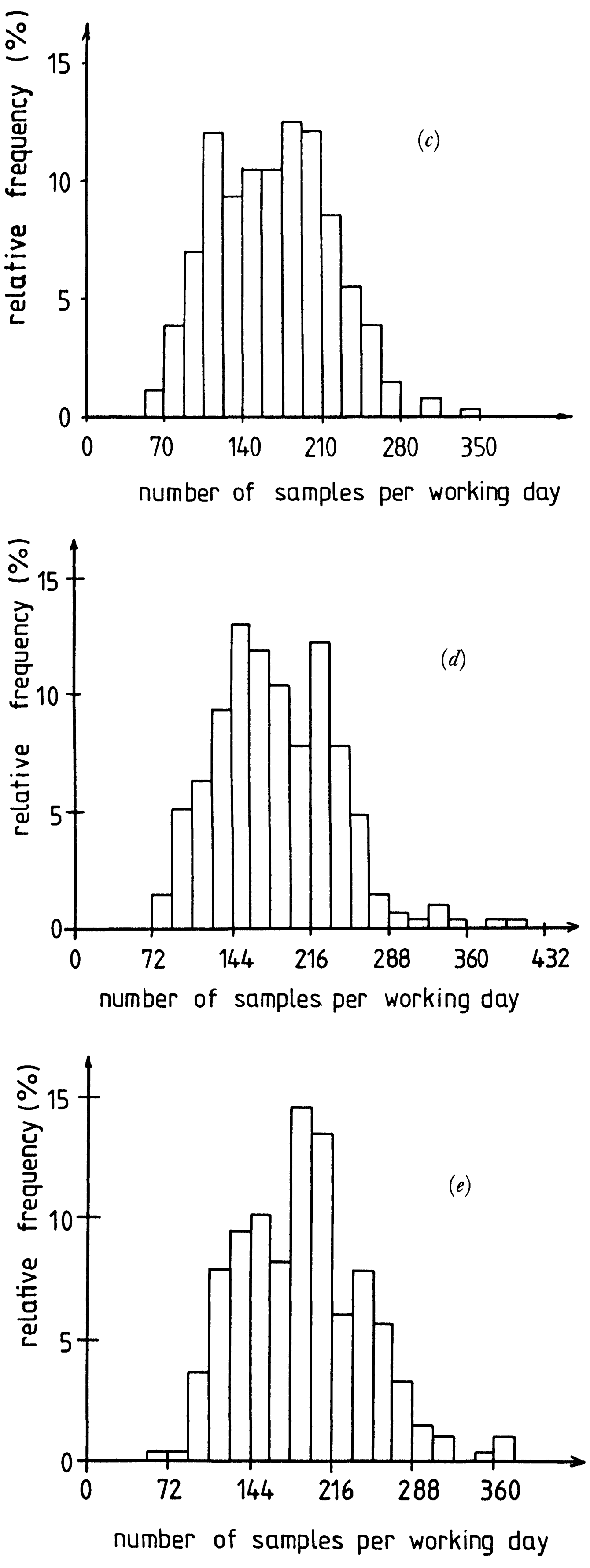

*Figure 1. The distribution of the number of blood samples per working day. (a) in 1980; (b) in 1981; (c) in 1982; (d) in 1983; and (e) in 1984.*

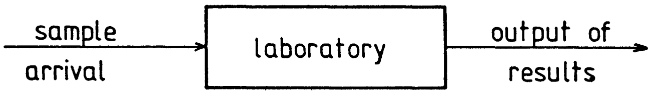

*Figure 2. The black-box model of the laboratory for the determination of alcohol-content of blood sample.*

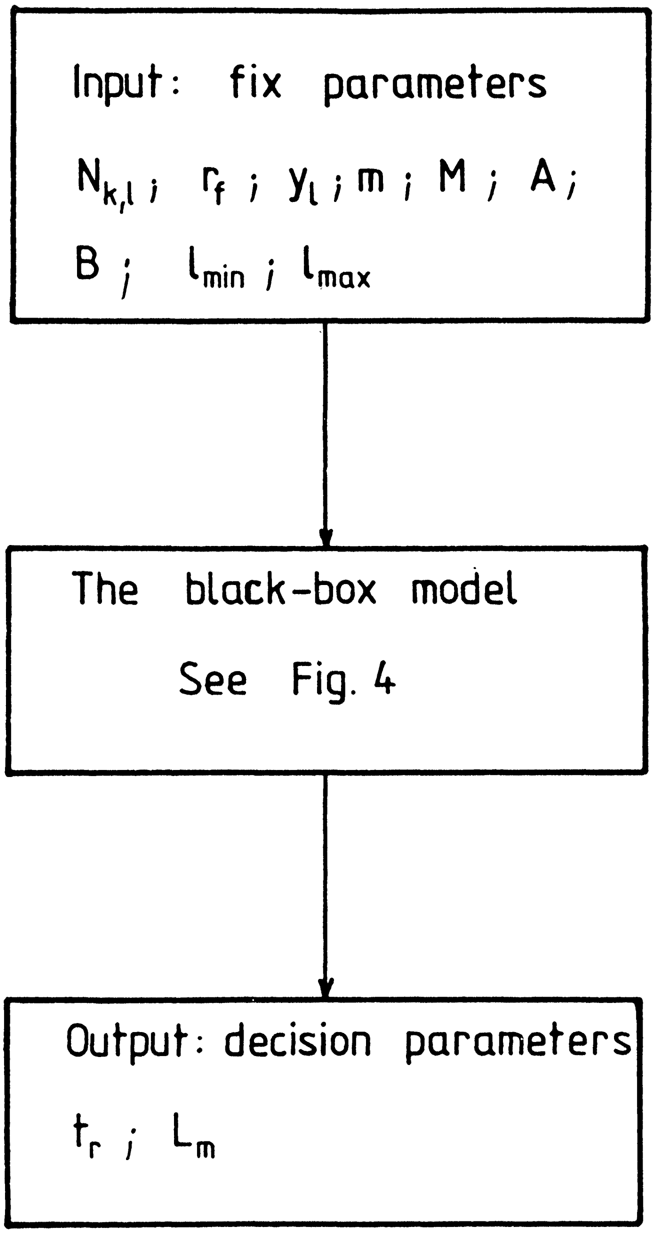

*Figure 3. Block-scheme of the computer program. Where $N_{k,l}$ = the number of blood samples with l priority, arrived in the kth working day of the tested year; $r_f$ = repetition factor ($r_f$ = 1·1); $Y_l$ = the number of blood samples with l priority arrived in the tested year; m = the tested smallest capacity in a working day; M = the largest possible value of m; A = 1, for calculation of running times; B = 1, for calculation of loading; $l_{min}$ = the smallest priority; $l_{max}$ = the largest priority; $t_r$ = running time; $L_m$ = loading at m samples/working day capacity.*

shown in figure 2. In the second stage, the working processes in the laboratory were divided into serials and parallel processes and the necessary capacity was determined in view of service times.

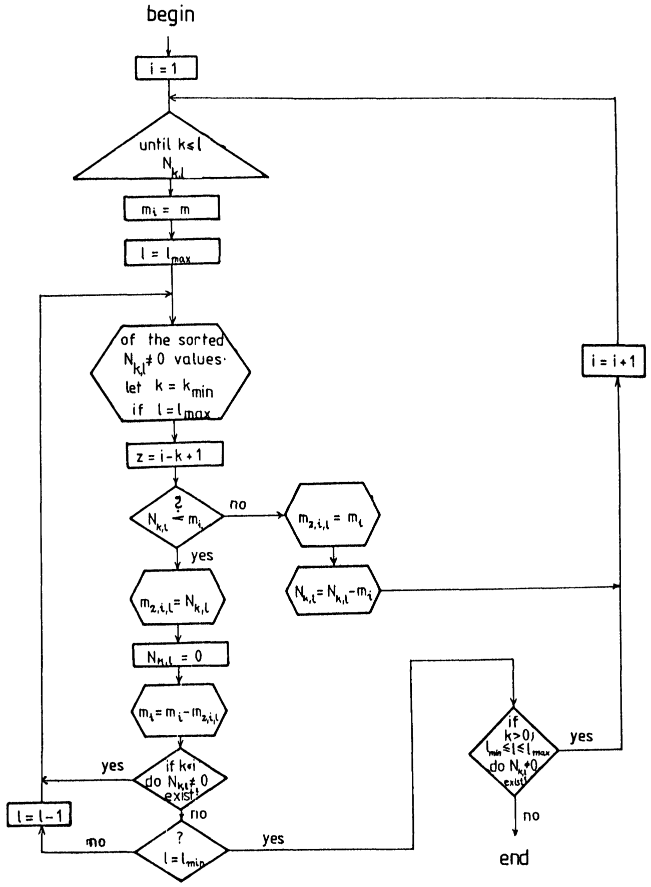

*Figure 4. Flow chart of the testing algorithm of the simplified queueing model of the laboratory. Where i = index, from 1 to $k_{max}$; $k_{max}$ = the number of working days in a year; k = the serial number of a working day; l = priority; $l_{max}$ = the largest priority; $l_{min}$ = the smallest priority; $N_{k,l}$ = the number of blood samples with l priority, arrived in the kth working day of the tested year; m = the tested smallest capacity in a day; $m_{z,i,l}$ = the number of blood samples with l priority, analysed within z working days on the ith working day.*

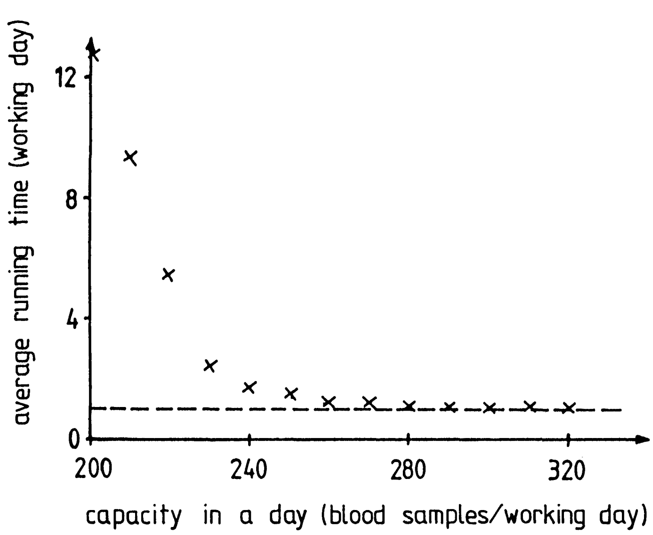

*Figure 5. The average running times in the function of the capacity in a working day on the basis of 1982's data.*

The numerical calculations can be reduced, when the service times are constant, because it is only necessary to consider the inter-arrival times. Calculations were made on the basis of the measured samples batched by day so differences in service times could be equalized. These expectations were later controlled experimentally.

Because of contradictions between analytical results and the medical symptoms of 10% of the measurements must be repeated. Repeated blood samples were considered as new samples, and the number of samples arriving was increased according to the ratio of repetitions.

Investigating the model discussed above is more simple, because the number of blood samples arriving in a working day are tested with different priorities in the function of the maximum measurable number of samples per working day. For such a model we can use a numerical method for the calculation in applying an appropriate algorithm. This solution is also applicable to inter-arrival times that are not independent.

In order to make the calculation the number of blood samples which had arrived in the previous years had to be transformed to the designed capacity of 50 000 samples per year. The transformed capacity in a working day was:

$$N_{i,k,l} = N_{k,l} \times r_f \times \frac{50\,000}{Y_l}$$

*Table 2. The probable running times at the capacity of 260 samples/working day and 50 000 samples/year.*

| Running time (days) | Ratio of the samples (%) |
|---|---|
| 1 | 77·4 |
| 2 | 21·8 |
| 3 | 0·8 |

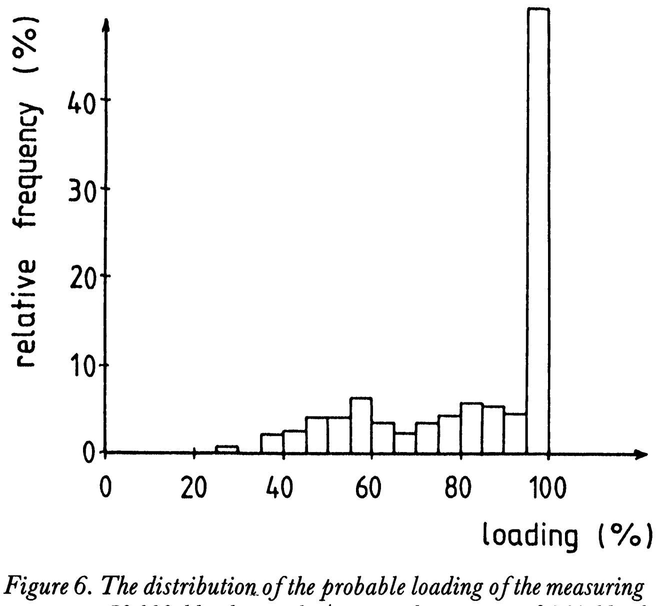

*Figure 6. The distribution of the probable loading of the measuring system at 50 000 blood samples/year and capacity of 260 blood samples/working day on the basis of 1982's data.*

where $N_{i,k,l}$ = the number of blood samples with priority $l$, transformed from the data of the tested year and probably arriving on the $k$ th working day (blood samples/working day).

$N_{k,l}$ = the number of blood samples with priority $l$, arriving on the $k$ th working day of the tested year.

$r_f$ = repetition factor ($r_f$ = 1·1).

$Y_l$ = the number of blood samples with priority $l$, arriving in the tested year.

On this basis an algorithm was elaborated describing the function of the simplified black-box model of the laboratory. Figure 3 shows the block-scheme of the computer program and figure 4 shows flow chart of the algorithm of the black-box model of the laboratory. The running times in the case of different measuring capacity per working day were calculated on the basis of the algorithm above from the data collected over the previous five years. For

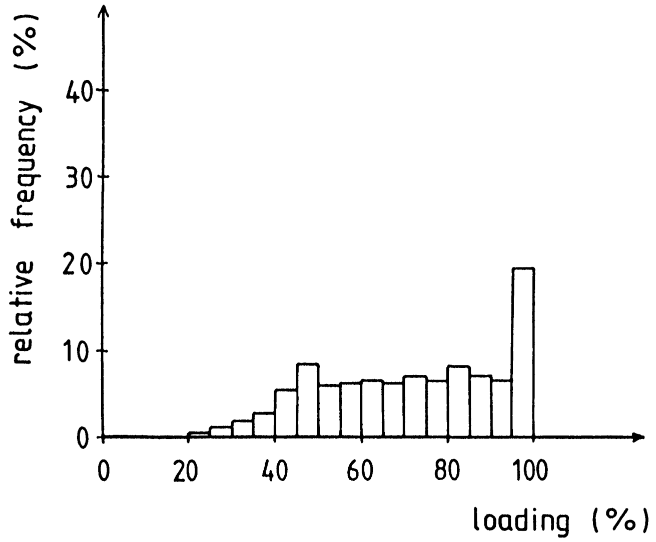

*Figure 7. The distribution of the probable loading of the measuring system at 50 000 blood samples/year and capacity of 300 blood samples/working day on the basis of 1982's data.*

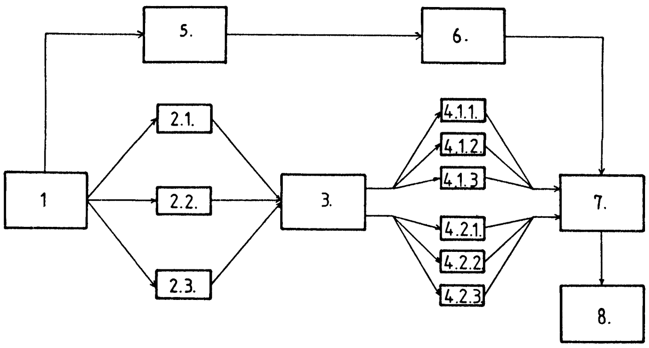

*Figure 8. The scheme of the organization of the designed laboratory. Where 1 = arrival of samples; 2.1 = the ith composition of the samples; 3 = the handling of instruments; 4.1.1 = the ith parallel analysis of the samples with odd serial number using headspace gas chromatographic method; 4.2.1 = the ith parallel analysis of the samples with even serial number using headspace gas chromatographic method; 5 = data input; 6 = data control and other administration; 7 = computerized data acquisition; and 8 = the quality control of analytical results.*

these five years very similar results were obtained, therefore we are showing a typical running time (figure 5).

It can be seen that at first the running times decrease exponentially with an increase in measuring capacity in a working day, and later there is no significant effect of the increasing capacity from the 240–260 blood samples/ working day. The obtained capacity must be increased with the number of the measurements for control of reliability of the analytical results. There are typically 20 measurements/instrument.

On the basis of the analysis of the running times it appears that it is not reasonable to increase capacity over 260 blood samples/working day, because the running time decreases only very slowly with capacity increases (see figure 5). The probable running times can be seen in table 2 at capacity of 260 blood samples/working day and 50 000 bloood samples/year.

The probable loading of the measuring channels was calculated, and their distributions can be seen in figures 6 and 7. The probable average loading of the laboratory is 84% in the case of two measuring channels and 260 blood samples per working day (figure 6). Comparing figure 7 with figure 6, it is evident that a 15% increase in capacity decreases effective laboratory performance: the number of working days loaded to 100% decreased to about one-third.

When the necessary total laboratory capacity is known, the necessary total capacity of internal processes of the laboratory is discovered: in further designs the incidental character of arrival of bloood samples should not then be taken into consideration.

The work of the laboratory was divided into internal processes, and the necessary times for performance of these processes were obtained by the help of model experiments. Only the results of this planning phase are shown: sample preparation needed 1·3 min, while the gas chromatographic analysis took 2 min. The data input to the computer of a sample takes about 1·4 min, the controlling data input is about 0·8 min, and all other administration takes 0·6 min. The time for real-time data acquisition is negligible.

The engineer controlling the work of the laboratory qualifies the analytical results with a computer, so this process ought not to be taken into account as a capacity determining factor. The running time seems to be as quick as possible, but at the same time there is the requirement to achieve the largest possible loading. As a result of these two contrasted effects the optimal running time is two days.

With these assumptions, the construction of the laboratory shown in figure 8 is considered optimal.

### Conclusions

An important use of queueing theory is the test of the independence of inter-arrival times. This is rarely done: more commonly, the numerical method based on the algorithm, which describes an appropriately simplified model of the laboratory is employed for calculating queueing parameters. Queueing theory helps to obtain a consistent description of a complex system but its usefulness is limited. The method proposed by the authors is based on the basic principles of the queueing theory and some simplified model and could lead to a better understanding and to the solution of very complex problems in the future.

### References

1. Kleinrock, L., *Queueing Systems. Volume I.: Theory* (John Wiley and Sons, Inc., New York, 1975).
2. Adeberg, V. and Doerffel, K., *Euroanalysis II* (Budapest, Hungary, 1975), abstracts of papers, p. 536.
3. Massart, D. L., Dijkstra, A. and Kaufman, L., *Evaluation and Optimization of Laboratory Methods and Analytical Procedures* (Elsevier Scientific Publishing Company, Amsterdam, 1978).
4. Vollenbroek, J. G. and Vandeginste, B. G. M., *Analytica Chimica Acta*, **133** (1981), 85.
5. Vandeginste, B. G. M., Digital simulation of a laboratory for structural analysis. Dissertation: University of Nijmegen, Toernooiveld, The Netherlands (1981).
6. Brownlee, K. A., Statistical theory and methodology. In *Science and Engineering*. 2nd edn (John Wiley and Sons, Inc., New York, 1965), 221–222.

